# Multicenter Genomic Analysis of Carbapenem-Resistant *Pseudomonas aeruginosa* in Austrian Community Hospitals Reveals Limited Carbapenemase Prevalence and Absence of Interhospital Clonal Spread

**DOI:** 10.3390/antibiotics15050516

**Published:** 2026-05-20

**Authors:** Magda Diab-Elschahawi, Tim Kirk, Susanne Häussler, Elisabeth Presterl, the PSPS Working Group

**Affiliations:** 1 Department of Hospital Epidemiology and Infection Control, Medical University, 1090 Vienna, Austria; magda.diab-elschahawi@meduniwien.ac.at; 2 Institute of Molecular Bacteriology, Twincore Centre of Experimental and Clinical Infection Research, a joint venture of the Hannover Medical School and the Helmholtz Centre for Infection Research, 30625 Hannover, Germanysusanne.haeussler@helmholtz-hzi.de (S.H.); 3 Department of Molecular Bacteriology, Helmholtz Centre for Infection Research, 38124 Braunschweig, Germany; 4 Department of Clinical Microbiology, Copenhagen University Hospital–Rigshospitalet, 2100 Copenhagen, Denmark

**Keywords:** *Pseudomonas aeruginosa*, multidrug-resistance, whole genome sequencing, transmission

## Abstract

**Background/Objectives**: In Europe, *Pseudomonas aeruginosa* is the second most common cause of ventilator-associated pneumonia in intensive care units. Intrinsic antibiotic resistance and acquired carbapenemases can lead to high mortality. To guide more targeted antimicrobial therapy and adequate infection control measures, we performed a multicenter study on the prevalence and genetic basis of carbapenem resistance among *P. aeruginosa* (CR-PA) across 17 community hospitals in Austria. **Methods**: During a 3-month period, we collected 621 *P. aeruginosa* isolates from 560 patients. Antibiotic susceptibility testing was performed according to EUCAST guidelines, and all CR-PA isolates were subjected to whole genome sequencing. **Results**: Antibiotic susceptibility testing revealed carbapenem resistance in 5.41% (36/621) of the investigated *P. aeruginosa* isolates. Only 3 produced a carbapenemase (2 Verona Integron-encoded Metallo- ß-lactamases and 1 Imipenemase Metallo-ß-lactamase) and carried a carbapenemase-encoding gene. Among the studied *P. aeruginosa* isolates there was a high genetic diversity, excluding a single driving epidemic lineage in the included Austrian hospitals. **Conclusions**: The absence of interhospital clonal dominance suggests that carbapenem resistance emerged independently in different centers, likely driven by local antibiotic selection pressures rather than regional clonal spread.

## 1. Introduction

According to biannual point prevalence surveys (PPS) on hospital-acquired infections (HAI) and antimicrobial use in acute care hospitals in Austria, about one-third of patients receive antimicrobials [1]. High antimicrobial consumption is often linked to the prevalence of specific resistant gram-negative bacteria, such as *P. aeruginosa* [2]. *P. aeruginosa*, a Gram-negative rod-shaped bacterium, is an opportunistic pathogen commonly found in moist environments. *P. aeruginosa* may colonize water systems not only in healthcare institutions but in any poorly maintained water infrastructure [3,4]. It may also colonize human mucous membranes, particularly those of the respiratory tract. Damage of the mucociliary system, e.g., in cystic fibrosis or chronic obstructive pulmonary diseases, enhances the building of complex biofilms and bacterial persistence despite antimicrobial treatment [5,6].

Patients with chronic respiratory disease, severe immunosuppression, including leukemia, cancer, and organ transplantation, severe burns, and polytrauma requiring long-term admittance to intensive care are at highest risk for acquiring a HAI with a multidrug-resistant *P. aeruginosa*, particularly in tertiary care hospitals [7]. HAI due to *P. aeruginosa* encompasses both life-threatening acute and persistent chronic biofilm-associated infections with limited treatment options, such as ventilator-associated pneumonia, wound infections, and catheter-associated urinary tract infections [8,9]. In Europe, *P. aeruginosa* is the second most common cause of VAP in intensive care units [1].

High intrinsic antibiotic resistance and acquired resistance against many antibiotics are of major concern, as they can limit the available treatment options, leading to high mortality [10]. Resistance mechanisms in *P. aeruginosa* include intrinsic chromosomally encoded class C (blaPDC) and D (blaOXA-50 family) β-lactamases, overexpression of efflux pumps, porin channel alterations, and the acquisition of additional β-lactamases, including carbapenemases, via horizontal gene transfer. While carbapenems are often used to treat infections caused by *P. aeruginosa*, resistance to these broad-spectrum antibiotics has been increasingly reported [1,2,3,4,5,6,7,8,9,10,11,12,13].

In Austria, 15% of invasive *P. aeruginosa* isolates tested are carbapenem-resistant [14]. There are several mechanisms of carbapenem resistance, including the production of carbapenemases [5,15,16]. Studies have shown that infections caused by carbapenemase-producing *P. aeruginosa* are associated with higher mortality and longer hospital stays. Carbapanenemases further limit the available treatment options, as newer antimicrobial agents such as ceftolozan-tazobactam, cefiderocol, and other antibiotic combinations are rendered ineffective. To use these new antimicrobials appropriately, knowledge about the prevalence of carbapenemases among carbapenem-resistant *P. aeruginosa* (CR-PA) will guide a more targeted, effective antimicrobial therapy, improving patient outcome.

Moreover, carbapenemases are frequently located on plasmids, which are mobile, horizontally transmissible genetic elements. From an infection prevention and control (IPC) perspective, the identification of such mobile carbapenemases among CR-PA is essential for appropriate action; i.e., to prevent their horizontal spread in the hospital setting [17]. However, because CR-PA isolates are not routinely screened for carbapenemases in many clinical laboratories, the exact proportion of carbapenemase-producing *P. aeruginosa* among CR-PA is unknown [17].

Although there are many studies on *P. aeruginosa* infections in tertiary care hospitals [7], little is known about the epidemiology and clonality of *P. aeruginosa* isolates in community hospitals. To investigate the prevalence and genetic basis of carbapenem resistance, we performed a prospective multicenter study of *P. aeruginosa* isolates from patients in community hospitals in different geographical regions of Austria, sequencing *P. aeruginosa* isolates to identify the acquisition of carbapenemase-encoding genes and explore potential associations with local antimicrobial usage. Specifically, we sought to assess: (a) the proportion of CR-PA among patients from whom *P. aeruginosa* was isolated from clinical cultures; (b) the proportion of carbapenemase-producing *P. aeruginosa* and the identification of carbapenemase-encoding genes among patients with CR-PA isolated from both screening and clinical cultures; (c) the antimicrobial susceptibility profiles of CR-PA to ceftolozane–tazobactam, ceftazidime–avibactam, cefiderocol, and imipenem–relebactam; (d) the clinical characteristics of patients with CR-PA isolates; and (f) the phylogenetic clustering patterns of the *P. aeruginosa* isolates based on whole-genome sequencing data.

## 2. Results

### 2.1. Description of the Hospitals Participating in the Study

In this prospective study, 34% (17/50) of invited Austrian network hospitals from our surveillance network participated, all of them community hospitals. The total number of beds across participating hospitals ranged from 106 to 1043, with a median of 233 beds. General care beds ranged from 100 to 1023 (median = 227). The number of intensive care beds varied between 0 and 40 (median = 6). Annual hospital admissions ranged from 4905 to 27,173 (median = 14,941). Patient days per year ranged from 19,548 to 247,947 (median = 60,142). The regional distribution of the participating hospitals comprised 7 of the 9 Austrian provinces (except for Vorarlberg and Burgenland). Of these, 5 (Salzburg, Styria, Tyrole, Upper Austria, and Vienna) supplied *P. aeruginosa* isolates and epidemiological data. Table 1 summarizes structural capacity and utilization data for the 17 participating Austrian hospitals.

Between April and June 2024, we received 751 *P. aeruginosa* isolates from 560 patients. Of those 751 isolates, only 621 were further analyzed in the laboratory (Figure S1: Selection workflow for *P. aeruginosa* isolates analyzed in the current study). Forty-three patients had more than one *P. aeruginosa* isolate with varying antimicrobial resistance patterns. Demographic characteristics and clinical information of the patients are summarized in Table 2. In total, 56.43% (316/560) of the patients were colonized with *P. aeruginosa*, while 43.57% (244/560) of patients had an infection with *P. aeruginosa*.

### 2.2. Results of Minimum Inhibitory (MIC) Concentration Testing of 181 Selected P. aeruginosa Isolates Are Shown in Table 3

MIC testing was performed in 181 *P. aeruginosa* isolates, results are given in Table 3.

**Table 3:** In vitro susceptibility testing results (MICs) for the selected 181 *P. aeruginosa* isolates.

*P. aeruginosa* Isolates (*n* = 181)	Ceftazidim	Cefepime	Imi-penem	Meropenem	Ciprofloxacin	Ceftolozane–Tazobactam	Ceftazidime–Avibactam	Cefiderocol	Imipenem–Relebactam
MIC 50	2	4	1	0.5	0.25	1	1	0.25	1
MIC 90	64	32	16	8	4	16	16	2	8
Range	1–64	0.25–64	0.5–64	0.25–128	0.25–4	1–64	1–64	0.03–64	1–64

### 2.3. Prevalence of Carbapenem-Resistant P. aeruginosa Isolates in a Network of Austrian Hospitals

Of the 621 *P. aeruginosa* isolates, 36 (5.41%) were carbapenem-resistant, and only 3 (0.48%) produced a carbapenemase; 2 Verona Integron-encoded Metallo-ß-lactamases (VIM) and 1 Imipenemase Metallo-ß-lactamase (IMP). A total of 36 (5.8%) were classified as 3 MRGN, showing resistance against three of the following antimicrobial agent groups (piperacillin, ceftazidim/cefepim, imipenem/meropenem, and ciprofloxacin). Demographic characteristics and clinical information of the 36 patients with CR-PA are summarized in Table 4. Of the patients, 61% (22/36) were colonized with CR-PA, while 39% (14/36) had an acute infection with CR-PA.

Among the 36 CR-PA isolates, 50% (18/36) were resistant against imipenem/relebactam, 8% (3/36) were resistant against cefiderocol, 44% (16/36) were resistant against ceftazidim/avibactam, and 45% (20/36) were resistant against ceftolozane/tazobactam. Among the 3 carbapenemase-producing CR-PA isolates, the 2 VIM-positive isolates were only sensitive to cefiderocol, while the single IMP-positive isolate was sensitive to all antimicrobials tested.

### 2.4. Phylogenetic Relationships Among P. aeruginosa Isolates Collected over a Defined Time Period Within a Network of Austrian Hospitals

**Figure 1:**
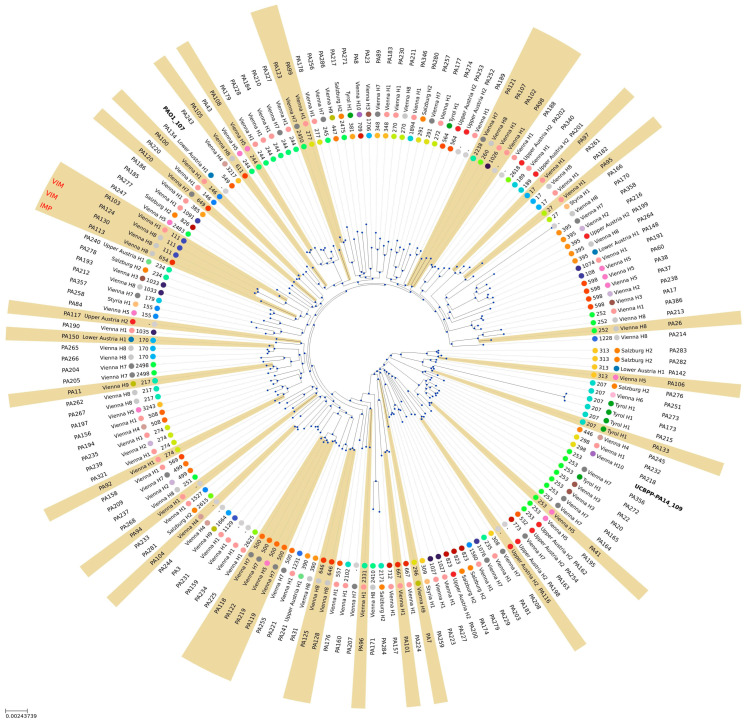
Phylogenetic tree of the sequenced *P. aeruginosa* strains and reference strains based on k-mer usage. If applicable, additional information about hospital of origin, MLST, and presence of carbapenemases (VIM, IMP) was added, and 4-MGRN isolates were indicated in light brown.

Of the 621 *P. aeruginosa* isolates included in this study, 181 were subjected to whole-genome sequencing, and only 165 of those were revealed to be *P. aeruginosa*. Figure 1 illustrates the phylogenetic relatedness of these 165 clinical isolates. We observed a broad distribution of *P. aeruginosa* MLST types across the population and the Austrian provinces, with carbapenem-resistant isolates dispersed throughout the phylogenetic tree.

**Figure 2:**
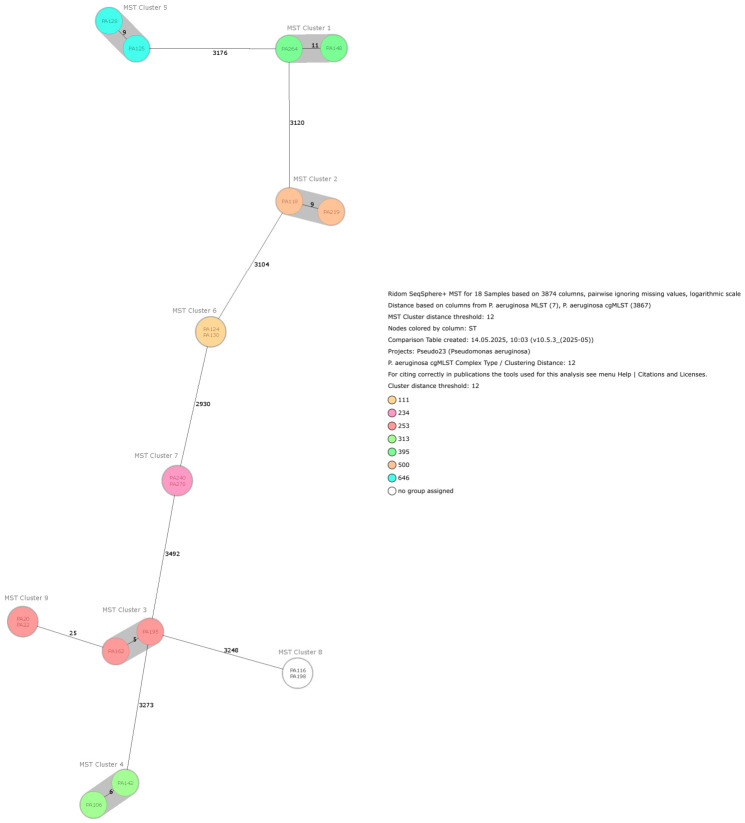
Minimum Spanning Tree for 19 *P. aeruginosa* samples based on 3874 columns, using pairwise distances while ignoring missing values and logarithmic scaling. The distance matrix was calculated using *P. aeruginosa* MLST (7 loci) and cgMLST (3867 loci). Sequence types (STs) and complex types (CTs) were assigned, with CTs used for cluster analysis and epidemiological investigation. WGS data interpretation and outbreak analysis were performed using Ridom SeqSphere+, applying a predefined cluster threshold of <12 allelic differences and constructing minimum spanning trees (MSTs). Nodes are colored according to Sequence Type (ST), and clusters are colored in grey.

Application of AMRFinderPlus on the whole-genome sequencing data confirmed the results of the immunochromatographic test and identified three isolates carrying acquired β-lactamases: two harboring a VIM and one carrying an IMP carbapenemase-encoding gene. The two VIM-positive isolates were phylogenetically closely related and originated from patients within the same hospital. In addition to the two closely related VIM-positive isolates, we identified eight further clusters using the SeqSphere+ software version 12, each comprising two closely related isolates. Figure 2 shows the minimum spanning tree, including SNP distances between and among these nine pairs of closely related isolates. Table S1 contains the MST cluster description including the number of patients per cluster (*n* = 2), isolate identification code within the MST cluster, sequence (ST) type, cluster (CT) type, type of hospital admission of the patient harboring the isolate (outpatient clinic = OC, general ward = GW, intensive care unit = ICU), and antibiotic resistance profile of the *P. aeruginosa* isolates. Clusters 6 (ST 111), 7 (ST 234), 8, and 9 (ST 253) always contained 2 patients harboring identical genotypes with no clustering distance at all, with patients from respective clusters 6, 8, and 9 being admitted to the same hospitals. MST cluster 6 (ST 111) contains the VIM-positive isolates. In contrast, patients belonging to cluster 7 had no geographical overlap within the same hospital during their admission. The two patients in cluster 5 (ST 146) were admitted to the same hospital within 6 days of each other, but were treated on different wards. Patients corresponding to clusters 1–4 (ST 395, 500, 253, and 313, respectively) were admitted to hospitals located in different provinces.

## 3. Discussion

Antimicrobial resistance to carbapenems among *P. aeruginosa* isolates from patients in Austrian community hospitals was low, particularly when compared with the 13.8% resistance rate reported by EARS-Net in 2024, which includes invasive isolates from hospitals nationwide [14]. Carbapenem use is a well-recognized driver of the emergence of carbapenem-resistant Enterobacterales [18]. Although antimicrobial stewardship programs have been shown to reduce overall antimicrobial consumption, their specific impact on carbapenem resistance remains uncertain [19]. In our study population, carbapenem resistance among *P. aeruginosa* isolates was low (5.41%), and carbapenemase genes were detected in only 0.48% of isolates. The molecular epidemiology analysis identified several internationally recognized *P. aeruginosa* high-risk clones, namely ST111, ST234, ST253, ST395, ST313, and ST146. These sequence types are of clinical and epidemiological interest because they are found to be associated with multidrug resistance, hospital outbreaks, persistence in healthcare environments, and the dissemination of carbapenemase genes [20].

Even though the detection of theses clones in some of our isolates supports the circulation of epidemic multidrug-resistant lineages in the study setting, overall, the sampled *P. aeruginosa* showed a high genetic diversity, excluding a single driving epidemic lineage in the included Austrian community hospitals. Only 4 of the 9 clusters were among patients of the same hospital. A nosocomial transmission is probable in at least one of the clusters, as the two patients were admitted to the same ward of the same hospital within 5 days of each other. In two other clusters, the two patients were admitted to the same hospital but on separate wards and at least one month apart, and the last cluster, taking place in the same hospital, concerned an outpatient and an admitted patient with no likely overlap, thus making nosocomial transmissions very unlikely. As our study also includes isolates with indistinguishable genetic profiles despite the absence of documented hospital overlap among affected individuals, this finding raises the possibility of transmission pathways beyond direct healthcare-associated contact. In particular, community-based sources or indirect transmission routes—such as asymptomatic carriers or contaminated surfaces—may contribute to the observed genetic clustering. Even though a more detailed investigation of these potential links would strengthen the interpretation of transmission dynamics, a limitation of our study is that our data does not allow for this further analysis. Outbreak investigations are generally carried out by the mandatory infection control teams, even in community hospitals. However, the presence of only two isolates, coupled with the delayed availability of comprehensive antimicrobial susceptibility testing in the routine setting, will draw attention but may not justify immediate action or an investigation.

All hospitals included in this study have established antimicrobial stewardship programs, and data from the most recent Austrian point prevalence survey in 2023 indicate that carbapenem consumption in community hospitals remains low compared with tertiary care centers. This restricted use likely contributes to the very low prevalence of carbapenemase-producing CR-PA observed in our cohort.

Overall, the coexistence of several high-risk clones in our study is an important finding because it reflects the circulation of internationally disseminated multidrug-resistant lineages with significant epidemic potential. However, epidemiologically linked cases were confined to individual hospitals, with no evidence of dominant clones spreading across multiple hospitals or Austrian provinces. The heterogeneous distribution observed in our dataset may reflect some local selective pressures, such as differences in antibiotic use and infection control practices. These results emphasize the importance of continuous molecular surveillance and infection-control strategies to limit the spread of these clinically relevant clones.

CR-PA presents a challenge when it comes to antibiotic treatment, and carbapenemase production even more so [10]. Carbapenemase production can significantly alter not only the efficacy of conventionally used antipseudomonal antibiotic agents but also the efficacy of newly introduced beta-lactam/beta-lactamase inhibitor combinations advertised for use against *P. aeruginosa*, such as ceftolozane–tazobactam, imipenem–relebactam, and ceftazidime–avibactam [21]. In our study, we found unexpected resistance to ceftolozane–tazobactam and ceftazidime–avibactam among the 36 CR-PA isolates. Thus, to ensure their continued efficacy, either routinely assessing the susceptibility of CR-PA to newly introduced β-lactam/β-lactamase inhibitor combinations or periodically assessing all *P. aeruginosa* isolates, particularly in community hospitals, is essential even within standard clinical settings. Due to improved diagnostic and therapeutic options, as well as the outsourcing of patients to outpatient care, we see a general increase in vulnerable patient groups among hospital admissions [7]. Therefore, infections due to CR-PA are likely to become more frequent. Treatment failure due to unrecognized antibiotic resistance among CR-PA will furtively increase, leading to early treatment failure and inevitably to rising infection control challenges pertaining to healthcare-associated infection transmission and multidrug-resistant microorganisms.

Knowing the percentage of carbapenemase producers among CR-PA is crucial for clinical, epidemiological, and public health reasons. It will help infection control personnel in hospitals identify high-risk isolates, leading to the implementation of enhanced infection control measures, including targeted screening and containment. Combining both phenotypic and genotypic laboratory methods may help shorten time to identify these multidrug-resistant microorganisms and improve therapeutic options. Additionally, early detection of clusters and transmission chains within and outside the hospital setting will improve infection transmission prevention and detect potential public health threats. Therefore, from a public health point of view, the surveillance of the proportion of Ccarbapenemase-positive CR-PA helps monitor regional and global trends, detect early emerging threats or outbreaks, and finally inform policy decisions on antimicrobial stewardship.

## 4. Materials and Methods

### 4.1. Hospital Recruitment

The National Reference Center for Healthcare-Associated Infections and Infection Control (NRCHAIIC) is hosted by the Department of Infection Control and Hospital Epidemiology of the Medical University of Vienna, which has been running the national Austrian Nosocomial Infection Surveillance System (ANISS) since 2008. As such, it is responsible for coordinating the biannual point prevalence survey (PPS) on HAI in acute care hospitals, compiling the data of approximately 100 participating Austrian hospitals according to protocols from the European Centre for Disease Prevention and Control (ECDC) [22]. All network hospitals were contacted to take part in this multicenter study using the ECDC PPS criteria for national sample representativeness. We aimed to include 30 community hospitals to achieve good coverage across Austria [23].

### 4.2. Patients and Samples

The microbiological laboratories of participating hospitals were asked to collect all *P. aeruginosa* isolates from any type of clinical specimen (e.g., rectal swab, blood, respiratory samples) obtained from included patients (inclusion criteria for the study were being a male or female patient aged 18–99 years) receiving in- or outpatient care at the respective hospital over a period of three months (April to June 2024). Only the first *P. aeruginosa* culture episode per patient was included for further analysis, unless additional isolates of the same patient showed newly acquired resistance patterns (e.g., development of AMR under antimicrobial therapy) on the basis of broth microdilution testing.

### 4.3. Clinical Epidemiological Data

All electronic medical records of patients with CR-PA were reviewed. For patients with an infection caused by CR-PA, the following additional data was recorded using a questionnaire: (1) age and sex, (2) McCabe score, (3) clinical specimen where CR-PA was detected (e.g., blood), (4) diagnosis, (5) comorbidities, (6) antimicrobial treatment, and (7) length of hospital stay and outcome (discharged alive or in-hospital death). The severity of disease was evaluated using the Mc Cabe Score [24].

### 4.4. Data Collection and Statistical Analysis

Each isolate was allocated a consecutive study number and thereby pseudo-anonymized. Different isolates pertaining to the same patient received an additional unique identifier (e.g., letters of the alphabet). The study number was noted on the questionnaire, and the microbiological sample sent to us. Results of microbiological analyses and additional clinical patient data, if an infection with CR-PA was present, were compiled in an excel sheet. Only authorized persons, i.e., the investigator and the study staff, had access to the original data. Only aggregated data was provided to the sponsor.

Descriptive statistical data analysis was performed using SPSS Statistics 31.0 (IBM Corp, Armonk, NY, USA).

### 4.5. Antimicrobial Resistance Analysis

*P. aeruginosa* isolates were identified using standard microbiological methodology. eSwab™ swabs (Copan Liquid Amies Elution Swab, Hain Lifescience GmbH, Nehren, Germany) were used to transport specimens. Prior to and following analysis, the *P. aeruginosa* isolates were preserved in a bacterial storage system (MastGroup Diagnostica GmbH, Reinfeld, Germany). Upon receipt, the samples were re-cultured and the species was confirmed by standard microbiological techniques.

Antimicrobial susceptibility testing was performed according to EUCAST guidelines [25]. EUCAST breakpoints were used to define resistance profiles and were interpreted to assign microorganisms to “Multidrug-resistant gram-negative (MRGN)” categories according to the criteria of the German Commission for Hospital Hygiene and Infection Prevention (KRINKO). This classification defines MRGN organisms as gram-negative bacteria resistant to three or four of four key antibiotic classes. Bacteria resistant to three (3MRGN) or four (4MRGN) of the following antibiotic groups were defined as MRGN: acylureidopenicillins (e.g., piperacillin), third-generation cephalosporins (e.g., cefotaxime or ceftazidime), fluoroquinolones (e.g., ciprofloxacin), and carbapenems (e.g., meropenem or imipenem). For MRGN classification, antimicrobial susceptibility testing of all isolates was performed using Mastdisks AST (MastGroup Ltd., Bootle, UK). Two Mueller–Hinton agar plates per isolate were used. The following antibiotic disks (concentration in µg; abbreviation) were tested: Plate 1 included piperacillin (30; PRL), ceftazidime (10; CAZ), cefepime (30; CPM), imipenem (10; IMI), meropenem (10; MEM), and ciprofloxacin (5; CIP). Further testing on plate 2 included ceftolozane–tazobactam (30/10; C/T), ceftazidime–avibactam (10/4; CZA), cefiderocol (30; FDC), and imipenem–relebactam (10/25; IMR). The plates were incubated aerobically at 35 ± 1 °C for 18 h. Inhibition zone diameters were measured in millimeters and interpreted according to EUCAST clinical breakpoints as susceptible (S), susceptible increased exposure (I), or resistant (R).

### 4.6. Minimum Inhibitory Concentration (MIC)

A total of 181 out of 621 clinical and screening non-repeat study isolates, including all CR-PA isolates, were selected for further MIC analysis. The selection process aimed to ensure that the sequenced subset represented the overall diversity of the study collection. Therefore, antimicrobial resistance profiles, as well as the regional and temporal distribution of the *P. aeruginosa* isolates, were taken into account when choosing isolates for sequencing. For the selected 181 isolates, 4MRGN (*n* = 36), 3MRGN (*n* = 37) and susceptible (*n* = 108), the susceptibility test was verified and completed by the MIC method, using MICRONAUT-S Labor Berlin MDR MIC-GN 2 microplates (SF-M/E1-349-100) and Mueller–Hinton Broth, cation-adjusted (CAMHB) (Bruker Daltonics GmbH & Co. KG, Bremen, Germany). Subsequently, the results were read and recorded using the microplate absorbance reader Sunrise (Tecan GmbH, Grödig, Austria). The following antibiotics were tested by MIC: amikacin, aztreonam, aztreonam/avibactam (RUO), cefepim, ceftazidim, ceftazidime/avibactam (RUO), tigecycline, ertapenem, fosfomycin, imipenem, imipenem/relebactam (RUO), meropenem, tigecycline, and tobramycin. For cefiderocol susceptibility testing, MIC determination was performed separately using UMIC Cefiderocol strips (UM-CID-040; Bruker Daltonics, Bremen, Germany) and iron-depleted CAMHB. Fresh bacterial colonies were suspended in sterile 0.9% saline and adjusted to 0.5 McFarland. Subsequently, the suspension was diluted in ID-CAMHB at a ratio of 1:200, and 100 µL of the inoculated broth were dispensed into each well of the UMIC strip. The strips were incubated in a humid environment under aerobic conditions at 35 ± 1 °C for 18 ± 2 h. MIC values were read visually.

### 4.7. Detection of Carbapenemase Production

Thirty-six isolates with increased MIC were analyzed for the presence of KPC, NDM, IMP, VIM, and OXA-48 carbapenemases. This analysis was conducted using the Carbapenem-resistant K.N.I.V.O. Detection K-Set (Lateral Flow Assay) (Era Biology Group, Tianjin, China).

### 4.8. Molecular Typing Using Whole Genome Sequencing (WGS)

The 181 out of 621 study isolates selected for the MIC analysis were further analyzed using WGS. Antimicrobial resistance gene detection was performed using AMRFinderPlus on assembled whole genome sequences. Specifically, de novo genome assemblies generated with Shovill were used as input for AMRFinderPlus, which identifies acquired resistance genes and relevant chromosomal resistance determinants by comparison against the curated NCBI AMR reference database. In more detail: DNA extraction, Hackflex library preparation, and 150 bp paired-end sequencing on NextSeq 500 or NovaSeq 6000 sequencing systems was performed as previously described [26]. Raw reads were subjected to adapter trimming and quality filtering using BBTools v38.93 [27] to remove sequencing adapters and low-quality bases. Taxonomic classification was performed with Kraken2 v2.1.2 [28], which applies an exact k-mer matching strategy against a reference database for read-level assignment, and only samples consistent with *P. aeruginosa* were retained for further analysis. Sequencing achieved a median coverage of 122× with approximately one million reads per sample. Additional quality control included assessment of assembly size (6.0–7.3 Mbp) and GC content (65–67%), consistent with reference *P. aeruginosa* genomes. Multilocus sequence types were determined using MLST v2.19.0 [29] with PubMLST schemes. De novo assemblies were generated using Shovill v1.1.0 [30], which integrates SPAdes [31] for short-read genome assembly. Genome annotation was performed using Prokka v1.14.6 [32] to predict coding sequences, ribosomal RNA genes, transfer RNAs, and additional genomic elements. The phylogeny of the sequenced *P. aeruginosa* strains was inferred from their assembled genomes using Mashtree v1.4.6 [33]. Tree visualization was performed with the ete3 toolkit v3.1.3 for python [34].

WGS data interpretation and outbreak analysis were additionally performed using Ridom SeqSphere+, applying a predefined cluster threshold of <12 allelic differences and constructing minimum spanning trees (MSTs).

## 5. Conclusions

Resistance to carbapenems due to the presence of carbapenemases is yet very low in Austrian community hospitals. The absence of interhospital clonal dominance suggests that carbapenem resistance emerged independently in different centers, likely driven by local antibiotic selection pressures rather than regional clonal spread. However, given the worldwide emergence of resistance to broad-spectrum antibiotics, easy availability of comprehensive susceptibility testing and periodical surveillance for the presence and types of carbapenemases in *P. aeruginosa* isolates are warranted as an early indicator of emerging antimicrobial resistance in community hospitals.

## Figures and Tables

**Table 1:** Summary of structural capacity and utilization data for 17 participating Austrian hospitals.

Hospital ID	Total Number of Beds	Number of General Care Beds/Intensive Care Beds	Admissions/Year	Patient Days/Year
A	183	177/6	8373	46,919
B	1043	1023/20	27,173	224,308
C	138	126/12	4905	24,499
D	280	274/6	16,044	60,142
E	233	227/6	9155	48,129
F	156	156/0	12,034	36,052
G	183	183/0	8792	45,699
H	284	254/16	12,845	76,728
I	226	220/6	15,765	54,928
J	382	366/16	17,677	101,131
K	645	625/20	23,494	152,237
L	106	100/6	5708	19,548
M	748	672/40	25,875	247,947
N	364	353/11	14,941	74,819
O	505	480/25	25,735	109,541
P	145	145/0	7351	32,127
Q	215	209/6	16,877	65,601

**Table 2:** Demographic data and clinical characteristics of the study population.

Variables	Patients (*n*)	Patients (%)
Gender		
male	306	54.64%
female	254	45.36%
Age		
<18	7	1.25%
18–24	17	3.04%
25–34	33	5.89%
35–44	29	5.18%
45–54	32	5.71%
55–64	54	9.64%
65–74	112	20%
75–84	182	32.5%
≥85	93	16.61%
McCabe Score		
Non-fatal	216	38.57%
Ultimately fatal	143	25.54%
Rapidly fatal	34	6.07%
unknown	158	28.21%
Patient receives antimicrobial therapy		
Yes	360	64.29%
no	200	35.71%

**Table 4:** Demographic data and clinical characteristics of the 36 patients with CR-PA isolates.

Variables	Patients (*n*)	Patients (%)
Gender		
Male	25	69.44%
Female	11	30.56%
Age		
18–24	0	0%
25–34	3	8.33%
35–44	5	13.89%
45–54	1	2.78%
55–64	4	11.11%
65–74	11	30.56%
75–84	9	25%
≥85	3	8.33%
McCabe Score		
Non-fatal disease	13	36.11%
Ultimately fatal disease	12	33.33%
Rapidly fatal disease	3	8.33%
unknown	8	22.22%

## Data Availability

The original data presented in this study have been deposited in GenBank with links to BioProject accession number PRJNA1452886.

## Supplementary Materials

The following supporting information can be downloaded at: https://www.mdpi.com/article/10.3390/antibiotics15050516/s1, Figure S1: Selection workflow for *Pseudomonas aeruginosa* isolates analyzed in the current study. Table S1: MST cluster description including number of patients.

## Abbreviations

The following abbreviations are used in this manuscript:

CR-PA	Carbapenem-resistant Pseudomonas aeruginosa
HAI	Hospital-acquired infection
IPC	Infection prevention and control
PPS	Point prevalence survey
VAP	Ventilator associated pneumonia
