# Supplementary material for: Multicenter Genomic Analysis of Carbapenem-Resistant Pseudomonas aeruginosa in Austrian Community Hospitals Reveals Limited Carbapenemase Prevalence and Absence of Interhospital Clonal Spread
Source: Antibiotics (Basel). 2026 May 20;15(5):516. doi: 10.3390/antibiotics15050516 (PMC13203791; doi:10.3390/antibiotics15050516)
Supplement: Supplementary file 1 [file antibiotics-15-00516-s001.zip › Figure S1 Legend to Classification tree Diab et al Anitibotics R1.pdf]

Figure S1: Classification tree of 165 *P. aeruginosa* isolates obtained from 751 initial *P. aeruginosa* isolates after antimicrobial resistance profiling and genetic analysis

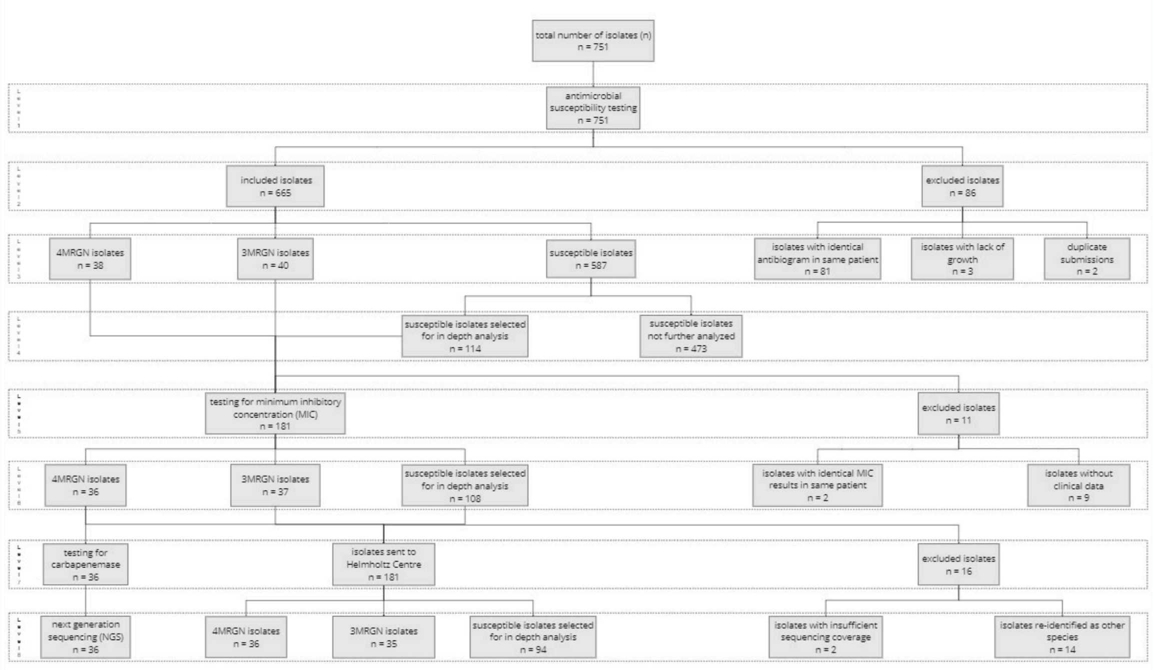

Figure S1 Legend: Classification tree of 165 *P. aeruginosa* isolates obtained from 751 initial *P. aeruginosa* isolates after stepwise antimicrobial resistance profiling and genetic analysis

A total of 751 *Pseudomonas aeruginosa* isolates were initially subjected to antimicrobial susceptibility testing in accordance with EUCAST guidelines. Following this, 665 isolates were retained for further analysis, while 86 were excluded due to identical antibiograms from the same patient (n = 81), lack of growth (n = 3), or duplicate submissions (n = 2). Based on their antibiograms, the 665 isolates were categorized into three groups: 4MRGN (n = 38), 3MRGN (n = 40) and susceptible (n = 587). From the latter group, 114 isolates were randomly selected, resulting in a total of 192 isolates subjected to minimum inhibitory concentration (MIC) testing. The remaining 473 isolates classified as susceptible *P. aeruginosa* were not further characterized.

Following MIC analysis, 11 isolates were excluded due to identical MIC results from the same patient (n = 2) or missing clinical data (n = 26), yielding a revised cohort of 181 isolates comprising 4MRGN (n = 36), 3MRGN (n = 37) and susceptible (n= 108) isolates. Carbapenemase testing using the Carbapenem-resistant K.N.I.V.O. Detection K-Set (Era Biology Group, China) was performed on the 4MRGN isolates. These isolates were subsequently analyzed by next-generation sequencing (NGS) using Illumina MiSeq technology. In addition, DNA from all 181 isolates was sent to the Helmholtz Centre for confirmation and phylogenetic analysis. Isolate selection for sequencing was designed to reflect the overall diversity of the study collection, taking into account antimicrobial resistance profiles as well as regional and temporal distribution.

Following external analysis at the Helmholtz Centre, an additional 16 isolates were excluded due to insufficient sequencing coverage (n = 2) or re-identification as other species (n = 14). This resulted in a final dataset of 165 *P. aeruginosa* isolates, comprising 4MRGN (n = 36), 3MRGN (n = 35) and susceptible (n = 94) isolates.
