# Supplementary material for: Multicenter Genomic Analysis of Carbapenem-Resistant Pseudomonas aeruginosa in Austrian Community Hospitals Reveals Limited Carbapenemase Prevalence and Absence of Interhospital Clonal Spread
Source: Antibiotics (Basel). 2026 May 20;15(5):516. doi: 10.3390/antibiotics15050516 (PMC13203791; doi:10.3390/antibiotics15050516)
Supplement: Supplementary file 1 [file antibiotics-15-00516-s001.zip › Table S1_MST Cluster Diab et al Antibiotics R1.pdf]

MST Cluster	Number of patients (n)	Isolate identification code	Sequence type (ST)	Complex Type (CT)	Type of admission: outpatient clinic, general ward or ICU	Antibiotic resistance profile (S=suceptible)
1	2	PA148<br>PA264	395<br>395	163<br>163	GW<br>GW	S<br>S
2	2	PA118<br>PA219	500<br>500	5742<br>5742	OC<br>GW/ICU	4MRGN<br>4MRGN
3	2	PA162<br>PA195	253<br>253	173<br>173	OC<br>GW	4MRGN<br>4MRGN
4	2	PA106<br>PA142	313<br>313	5737<br>5737	OC<br>GW	S<br>4MRGN
5	2	PA125<br>PA128	146<br>146	5732<br>5732	ICU<br>GW	4MRGN<br>4MRGN
6	2	PA124<br>PA130	111<br>111	2226<br>2226	ICU<br>ICU	4MRGN (VIM)<br>4MRGN (VIM)
7	2	PA240<br>PA278	234<br>234	6682<br>6682	GW<br>GW	4MRGN<br>S
8	2	PA116<br>PA198	n.a.<br>n.a.	5740<br>5740	OC<br>GW	4MRGN<br>3MRGN
9	2	PA20<br>PA22	253<br>253	70<br>70	GW<br>GW	S<br>S

Table S1: MST cluster description including number of patients per cluster (n=2), isolate identification code within the MST cluster, sequence (ST) type, cluster (CT)-type, type of hospital admission of the patient harboring the isolate (outpatient clinic = OC, general ward = GW, intensive care unit = ICU) and antibiotic resistance profile of the *P. aeruginosa* isolates
